# Twenty‐four hour continuous transvenous temporary right ventricular pacing in healthy horses

**DOI:** 10.1111/jvim.17027

**Published:** 2024-03-21

**Authors:** Amanda Avison, Anna R. Gelzer, Virginia B. Reef, Kathryn B. Wulster Bills, Cris Navas de Solis, Marc S. Kraus, JoAnn Slack, Darko Stefanovski, Lindsay J. Deacon, Claire Underwood

**Affiliations:** ^1^ Department of Clinical Studies, New Bolton Center School of Veterinary Medicine, University of Pennsylvania Kennett Square Pennsylvania USA; ^2^ Department of Clinical Sciences and Advanced Medicine School of Veterinary Medicine, University of Pennsylvania Philadelphia Pennsylvania USA

**Keywords:** atrioventricular block, cardiac, echocardiography, equine, syncope fluoroscopy

## Abstract

**Background:**

The ability to perform transvenous temporary cardiac pacing (TV‐TP) is critical to stabilize horses with symptomatic bradyarrhythmias. Reports of successful TV‐TP in horses are limited, and only briefly describe short‐term pacing.

**Objective:**

To describe temporary, medium‐term (24 h) transvenous right ventricular pacing in awake horses using a bipolar torque‐directed pacing catheter.

**Animals:**

Six healthy adult institutional teaching horses.

**Methods:**

Prospective experimental study with 2 immediately successive TV‐TP lead placements in each horse with a target location of the RV apex. One placement was performed primarily with echocardiographic guidance and 1 primarily with fluoroscopic guidance. In all placements, corresponding images were obtained with both imaging modalities. Horses were then paced for 24 h, unrestricted in a stall with continuous telemetric ECG monitoring. Echocardiographically determined lead position, episodes of pacing failure in the preceding 6 h, and pacing thresholds were recorded every 6 h. Pacing failure was defined as a period of loss of capture longer than 20 s.

**Results:**

Pacing leads were placed with both guidance methods and maintained for 24 h with no complications. Two horses with leads angled caudally in the right ventricular apex had no pacing failure, the remaining 4 horses had varying degrees of loss of capture. Leads located in the right ventricular apex had longer time to pacing failure and lower capture thresholds *P* < 0.05.

**Conclusions and Clinical Importance:**

Medium‐term TV‐TP is feasible and has potential for stabilization of horses with symptomatic bradyarrhythmias. Lead position in the right ventricular apex appears optimal. Continuous ECG monitoring is recommended to detect pacing failure.

**Abbreviations**
LOC: loss of capture
ROM: range of motion
RV: right ventricle
RVOT: right ventricular outflow tract
TV‐TP: transvenous temporary cardiac pacing

## 1 INTRODUCTION

Cardiac pacing is the treatment of choice for symptomatic bradyarrhythmias in humans^1^, ^2^, ^3^, ^4^, ^5^, ^6^, ^7^, ^8^, ^9^ and dogs.^10^, ^11^, ^12^, ^13^, ^14^ In equids, permanent cardiac pacing has been reported for third‐degree atrioventricular block, sick sinus syndrome, advanced second‐degree atrioventricular block, sinus arrest, and marked sinus bradycardia.^15^, ^16^, ^17^, ^18^, ^19^, ^20^, ^21^, ^22^ Horses presenting with these bradyarrhythmias frequently show signs of poor perfusion, are unstable, and at risk of cardiac failure and death.^17^, ^19^, ^23^, ^24^ This is distressing to owners and presents a safety issue to veterinary staff. Treatment with anti‐inflammatories, parasympatholytics, and/or sympathomimetics is often unsuccessful,^21^, ^25^, ^26^, ^27^, ^28^, ^29^, ^30^, ^31^ leaving cardiac pacing the only therapeutic intervention. Permanent pacemaker implantation is often not immediately available because of numerous considerations, including cost and the possible requirements for general anesthesia or heavy sedation, highly trained personnel, and specialized equipment.^15^, ^16^, ^19^, ^22^ In contrast, the equipment for transvenous temporary cardiac pacing (TV‐TP) is inexpensive and readily obtained. There are few reports of TV‐TP in equids. These briefly describe short‐term (<4 h) TV‐TP during permanent pacemaker implantation,^15^, ^16^, ^19^, ^22^ or for treatment of transient asystole after electrical cardioversion under general anesthesia.^32^ Reported challenges of TV‐TP in horses include maintaining electrode position and achieving consistent capture.^18^, ^24^

Medium‐term TV‐TP could be used to stabilize equids with symptomatic bradyarrhythmias, reducing the risk of injury to the animal and personnel. A period of stabilization with TV‐TP provides time for logistical arrangements and an informed discussion with the owner regarding treatment options. Maintenance of a normal ventricular rate (and myocardial perfusion) during a period of temporary pacing may also facilitate return to normal rhythm without the need for permanent cardiac pacing.^24^ Temporary cardiac pacing leads also have potential in horses considered at increased risk for development of bradyarrhythmias during sedation and general anesthesia. Whereas, echocardiography is typically used to guide placement of pacing devices in equids,^18^, ^20^, ^24^ fluoroscopic guidance is the standard for implantation of transvenous pacing devices in other species.^5^, ^33^, ^34^ Fluoroscopic guidance has historically been limited in horses because of their large body side. However, recent advances in robotic‐controlled imaging systems introduce the possibility of standing fluoroscopic guidance of temporary pacing devices.

The objectives of this study were to (1) establish whether transvenous pacing leads could be placed and maintained in the right ventricle (RV) of healthy horses for 24 h, (2) describe TV‐TP placement technique under both echocardiographic and standing fluoroscopic guidance (3) determine whether there are any complications associated with the procedure, and (4) identify factors for further investigation to improve pacing success.

## 2 MATERIALS AND METHODS

### 2.1 Animals

Six university‐owned horses, aged 6–30 years, were enrolled in this prospective experimental study. Body weights ranged from 470 to 655 kg and breeds were 3 Thoroughbreds, 1 Warmblood, 1 Standardbred, and 1 Quarter Horse. All horses were systemically healthy with normal cardiac rhythm. A preprocedural echocardiogram demonstrated normal cardiac chamber size and function with no significant valvular regurgitation. The project was approved by the Ethical Committee of the Veterinary School of University of Pennsylvania (IACUC #806884).

### 2.2 Study Design

**FIGURE 1:**
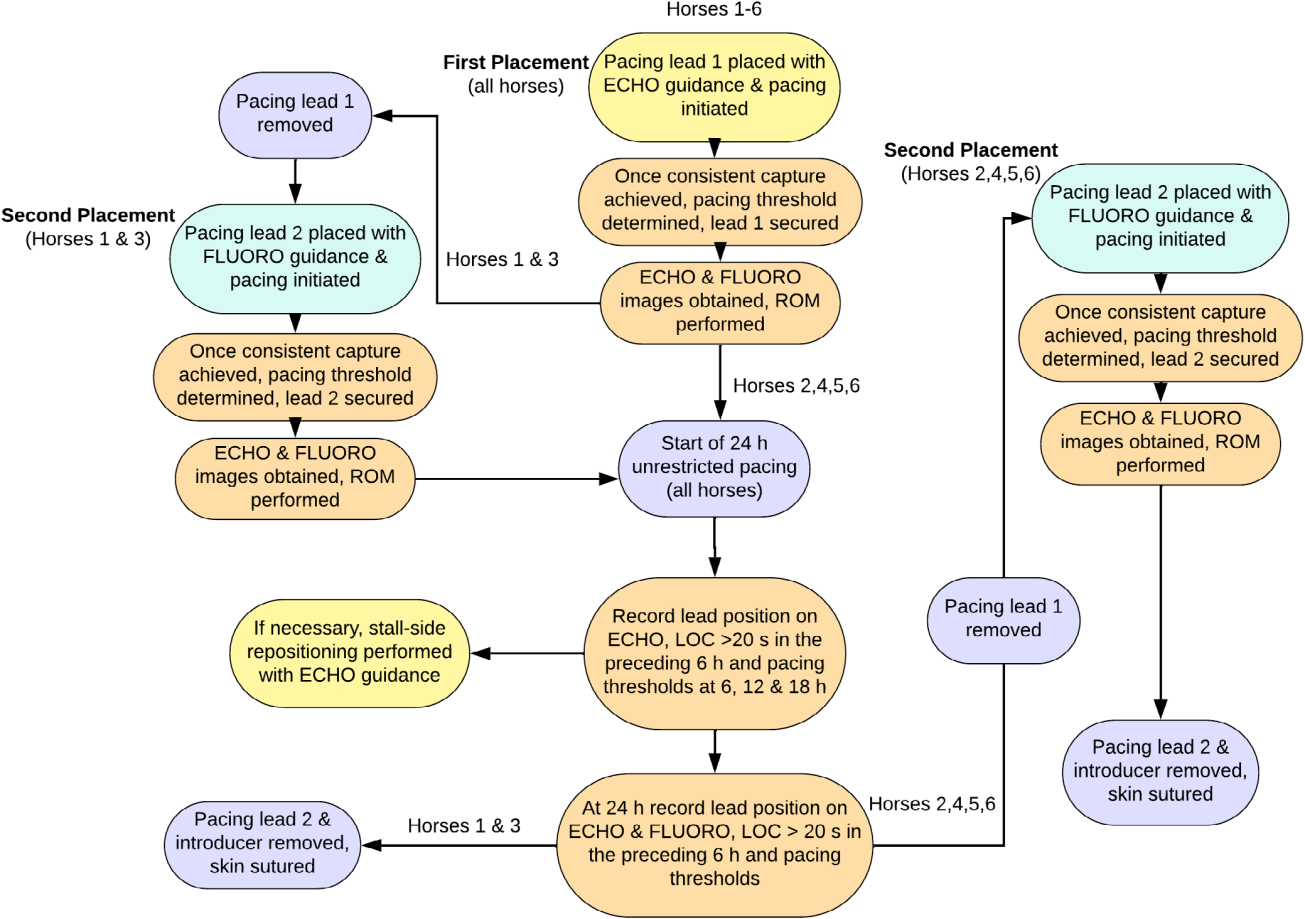
Flowchart showing first and second pacing lead placements in all horses relative to the 24 h monitoring period. ECHO, echocardiographic; FLUORO, fluoroscopic; LOC, loss of capture; ROM, range of motion.

A schematic diagram summarizing the study design is shown in Figure 1. Briefly, transvenous pacing leads were placed twice in each horse during standing sedation, initially primarily with echocardiographic guidance (GE Vivid E95 with M5S phased array transducer. Waukesha, WI, USA). After this the lead was removed and a second lead was placed primarily using fluoroscopic guidance (Figure S1A, VarexB147H housing/G892 insert, Varian4343CB detector. Salt Lake City, UT, USA). The RV apex was the target lead location for all placements. In each horse, a single pacing lead was maintained for a 24 h pacing period when the horses were unrestricted in stalls. The unrestricted pacing period occurred after placement primarily with echocardiographic guidance in 4 horses and fluoroscopic guidance in 2 horses.

### 2.3 Lead placement

A 14G intravenous catheter was placed aseptically in the right jugular vein. All horses were sedated as needed with a combination of IV acepromazine, butorphanol, and detomidine. Flunixin meglumine (1.1 mg/kg IV q24h) was administered for 2 days starting the morning of initial lead placement. Prophylactic antimicrobials (procaine penicillin (22 000 IU/kg IM q12h), and gentamicin (8 mg/kg IV q24h) were also administered for 2 days in 3 horses. A telemetric ECG (Televet Telemetric ECG version 7.0. Engel Engineering Service GmbH, Heusenstamm, Germany) for real‐time monitoring, and a Holter ECG (Pathfinder SL, Spacelab's Healthcare, WI, USA) for 24 h monitoring were both applied using a previously published modified base‐apex configuration.^35^ The telemetric ECG artifact and power supply filters were disabled to allow visualization of pacing spikes.

A 7‐Fr 11 cm Flexsheath introducer with hemostatic valve (Teleflex Medical, IDA Business Park, Ireland) was placed aseptically in the caudal third of the left jugular vein using standard technique. A 2‐electrode 110 cm 6‐Fr bipolar torque‐directed pacing catheter (Teleflex Medical) with electrode width of 2 mm and electrode spacing of 10 mm was passed by a single operator (AA) using aseptic technique.

The pacing catheter lead was passed through the introducer without venous distension until visualized in the right atrium. The lead was advanced into the RV, with adjustments to lead position (rotations, retractions, and advancements) performed either under echocardiographic guidance at the discretion of the echocardiographer (CU), or under fluoroscopic guidance at the discretion of the radiologist (KW). The lead was manipulated until the desired position was achieved in the RV apex,^15^, ^16^ or until capture was consistent in either the mid RV or right ventricular outflow tract (RVOT) if the RV apex position could not be achieved. Once positioned in the RV, the lead was attached to a temporary single chamber external generator (Medtronic temporary external pacemaker 5392. Medtronic, Minneapolis, USA) and synchronous pacing initiated in VVI mode with a rate of 60 beats/min to overdrive the intrinsic cardiac rhythm. Output and sensitivity were initially set at 10 mA and 2.0 mV, respectively. Once consistent pacing was achieved, the pacemaker capture and sensitivity thresholds were determined as previously described.^5^ The sensitivity was set at half the sensing threshold value and the output was set at double the capture threshold value per manufacturers recommendations. The lead was contained within a catheter sheath to maintain sterility and sutured in place near the hemostatic valve of the introducer sheath with a Tuohy‐Borst adapter (Cook Medical LLC, Bloomington, USA; Figure S1B) to facilitate repositioning. The time required for lead placement was recorded as the time from passing through the introducer until it was sutured in place.

Regardless of the primary imaging modality used, a set of comparable images were obtained with the alternate modality once the pacing lead was in position. Cine‐loops of the following echocardiographic right parasternal views were acquired: 4 chamber, left ventricular outflow tract, and RVOT from the fourth intercostal space and the RV apex in short and long axis from the fifth intercostal space. Additional modified views were acquired to follow the path of the lead as indicated. Using a robotic controlled imaging system, fluoroscopic left to right lateral cine‐loops focusing on the cardiac apex, with a maximum technique of 125 kV and 20 mA and automatic exposure control were acquired and stored, documenting the position. Collimation was used ad libitum if there was poor visualization of the lead. Repositioning was performed if deemed necessary based on the views of the secondary imaging modality with the aim of optimizing position in the RV apex. When repositioning was performed with the secondary modality, corresponding images of the new lead position were obtained with the primary imaging modality so all placements had complementary sets of echocardiographic and fluoroscopic images.

Range of motion (ROM) exercises were then performed including head stretches and walking tight circles in each direction. If capture was not consistent, or the lead moved during ROM, the lead was repositioned until consistent capture and position were achieved during ROM. A final set of fluoroscopic and echocardiographic images were obtained after ROM exercises. Lead length inserted was estimated based on marks every 10 cm from the tip to the location where the catheter entered the introducer sheath.

### 2.4 Unrestricted pacing period

A nonsterile stockinette neck wrap was applied to cover the catheters and the pacemaker was secured to a leather surcingle (Figure S1C). The horse was returned to its stall where it was allowed unrestricted movement with ad libitum access to hay and water for 24 h. During this time, the telemetric ECG was monitored remotely each hour for successful capture and arrhythmias. If capture was inconsistent, pacemaker settings were adjusted and/or leads were repositioned stall‐side using echocardiography. Every 6 h, the previously described echocardiographic views (GE Vivid IQ with 3Sc‐RS cardiac sector probe. Waukesha, WI, USA), episodes of pacing failure in the preceding 6 h, and pacemaker thresholds were documented. At 24 h, a corresponding set of fluoroscopic images were also obtained before lead removal.

At the conclusion of the study period, the pacing lead and introducer were removed together. A single skin suture with 2‐0 nylon suture (Ethicon, Ethicon Inc., Somerville, New Jersey, USA) was placed. The horses were closely monitored in their stalls for 24 h after pacing lead removal. This included continuous ECG assessment for the presence of arrhythmias. A complication was recorded if it resulted in clinical signs or necessitated treatment. The horses underwent an echocardiogram and 24 h ECG 1–3 months after the procedure and have since remained as institutional teaching animals.

Cardiac troponin I was measured on lithium heparinized plasma obtained before pacing lead placement (baseline) and at 6, 24, and 48 h after pacing lead placement using a previously validated, conventional assay (Stratus CS STAT Fluorometric Analyzer, Siemens Healthcare Diagnostics, Newark, USA).^36^

### 2.5 Image analysis

**FIGURE 2:**
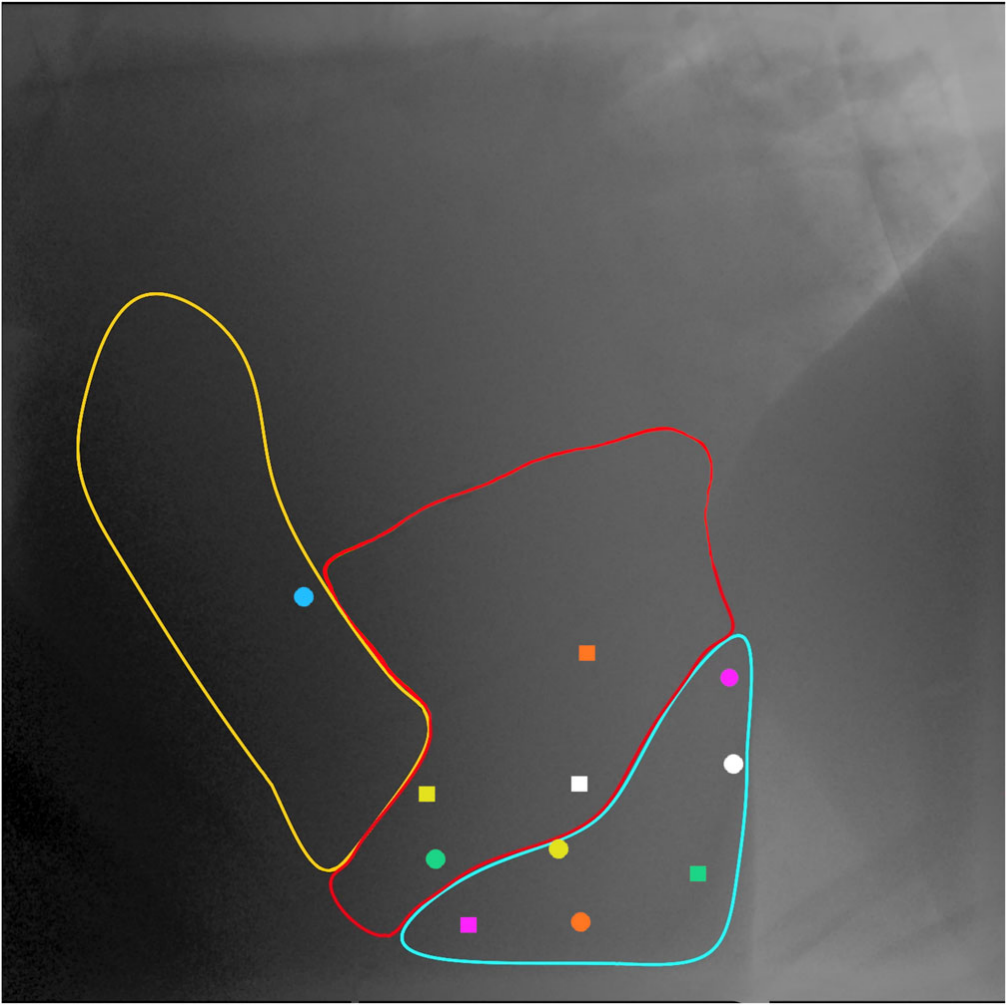
Right lateral fluoroscopic image outlining the different lead positions after each placement. The blue area corresponds to the right ventricle (RV) apex; the red area corresponds to the mid RV; and the yellow area corresponds to the RV outflow tract. The circles represent echocardiographic guided placements and squares fluoroscopic guided placements. Each color represents a different horse. Cranial is to the left of the image.

Echocardiographic and corresponding fluoroscopic images obtained at placement, repositioning, after ROM studies and at the end of the 24 h pacing period were reviewed simultaneously by the echocardiographer (CU) and the radiologist (KW) and categorized retrospectively using the lead position categorizations: (1) RV apex, (2) Mid RV, and (3) RVOT. The categorization was not blinded as both modalities were used to determine lead position to assist in interpretation of fluoroscopic images. Echocardiographic positions were defined as follows: (1) RV apex position—visible from the fifth intercostal space wedged in the RV apex, (2) mid RV—lead tip at or proximal to the moderator band 3) RVOT position—lead tip visible in the RVOT. Fluoroscopic positions (Figure 2) were categorized based on experience performing fluoroscopy on an equine postmortem heart with the pacing lead in different positions (unpublished data), extrapolation from canine images,^37^ and input from concurrent echocardiographic images. The pacing lead configuration on echocardiography and fluoroscopy was also categorized independent from position as: angled/curved cranially, angled/curved caudally, or straight. Echocardiographic images acquired at 6, 12, and 18 h were also reviewed and classified according to the above scheme.

### 2.6 ECG review and measures of pacing success

ECG files were analyzed retrospectively to determine pacing success using inbuilt software. Paced beats and sinus beats occurring at a rate above the pacing rate of 60 bpm were automatically marked as complexes by the software, whereas sinus beats that occurred at a rate below 60 bpm were manually removed and considered loss of capture (LOC) periods. Three measures of pacing success were calculated: (1) time to first LOC period >20 s (LOC > 20 s), (2) longest LOC period in each timepoint, and (3) average number of LOC > 20 s per hour. A LOC length of 20 s was chosen as being clinically significant based on previous reports in horses of ventricular pauses of 18–20 s or more resulting in syncope.^24^, ^27^

Telemetric ECG files were retrospectively analyzed at both placements and each of the 6 h timepoints for QRS morphology, peak amplitude, and QRS width of paced beats. Using the aforementioned modified base‐apex configuration channels 1, 2, and 3 correspond to modified base‐apex leads 1, 2, and 3, respectively. Peak amplitude and QRS width were measured on 3 consecutive paced beats and averaged. QRS morphology was characterized as previously described^38^; in brief, the wave of the largest amplitude was given a capital letter and other waves were assigned lower‐case letters. Waves were considered deflections of more than ±0.1 mV from the baseline.

### 2.7 Statistical analysis

Statistical analyses were performed using commercially available software (GraphPad, Boston, USA), and STATA (StataCorp LLC, College Station, USA). Descriptive statistics for continuous data are reported as medians [interquartile range] or mean ± SD [95% CI] as appropriate. Data were assessed for normality using Shapiro‐Wilk tests. Using data from placement and each of the 6 h check points 2 null hypotheses were tested: (H1) There is no difference in measures of pacing success (time to LOC > 20 s, number of LOC > 20 s/h, and longest LOC) at the target position of the RV apex compared with other positions and (H2) there is no difference in capture threshold at the target position of the RV apex compared with other positions. Multilevel mixed effects linear regression was performed with “time to LOC > 20 s,” “number of LOC > 20 s/h,” “longest LOC,” and “capture threshold” set as the outcome variables, the fixed effect as the target position of the RV apex, primary guidance modality set as a confounder and random effects set on the level of the individual animal with robust estimation of the variance. Post hoc analyses were performed to determine marginal (model adjusted) means and effects. To correct for multiple comparisons the least significant difference method was used. In horse 5, at the 12–18 h timepoint, the Tuohy‐Borst adapter used to secure the lead loosened resulting in retraction of the lead by 3 cm, possibly causing LOC. Hence, this datapoint was removed from analyses of pacing success (H1), but included for the analysis of capture threshold (H2) because this was measured at 12 h before the lead moved. Results are reported as marginal (model adjusted) means and 95% confidence intervals (95% CI). To avoid any confusion and for marginal means that were not different than zero, the lower 95% CI limit was set to 0 instead of the identified negative value. Statistical significance is reported as *P* < 0.05.

## 3 RESULTS

Transvenous pacing leads were placed with both echocardiographic and fluoroscopic guidance and maintained for a 24 h period in all 6 horses. Repositioning after initial placement was performed with the secondary guidance modality in 3/6 placements for each imaging modality. Figure 2 shows the final fluoroscopic lead positions after each placement. Pacing lead placement time was 38 ± 12 (25–51) min for leads placed primarily with echocardiographic guidance and 27 ± 13 (13–40) min for leads placed primarily with fluoroscopic guidance. When considering time for all placements (echocardiographic‐ and fluoroscopic‐guided), initial placement took 32 ± 13 (24–41) min. This increased to 44 ± 22 (30–59) min when time for repositioning with the secondary imaging modality was included. The distance from the catheter tip to where it enters the introducer was 80 ± 6 (78–82) cm. Lead position was adjusted due to lead movement and/or loss of capture during ROM in 3/12 placements. After these repositionings, all horses had consistent capture and stable lead position through subsequent ROM exercises. Because of the lack of available information and experience with TV‐TP in horses, slight variations in technique occurred to address challenges encountered, these variations are outlined in Table S1.

**FIGURE 3:**
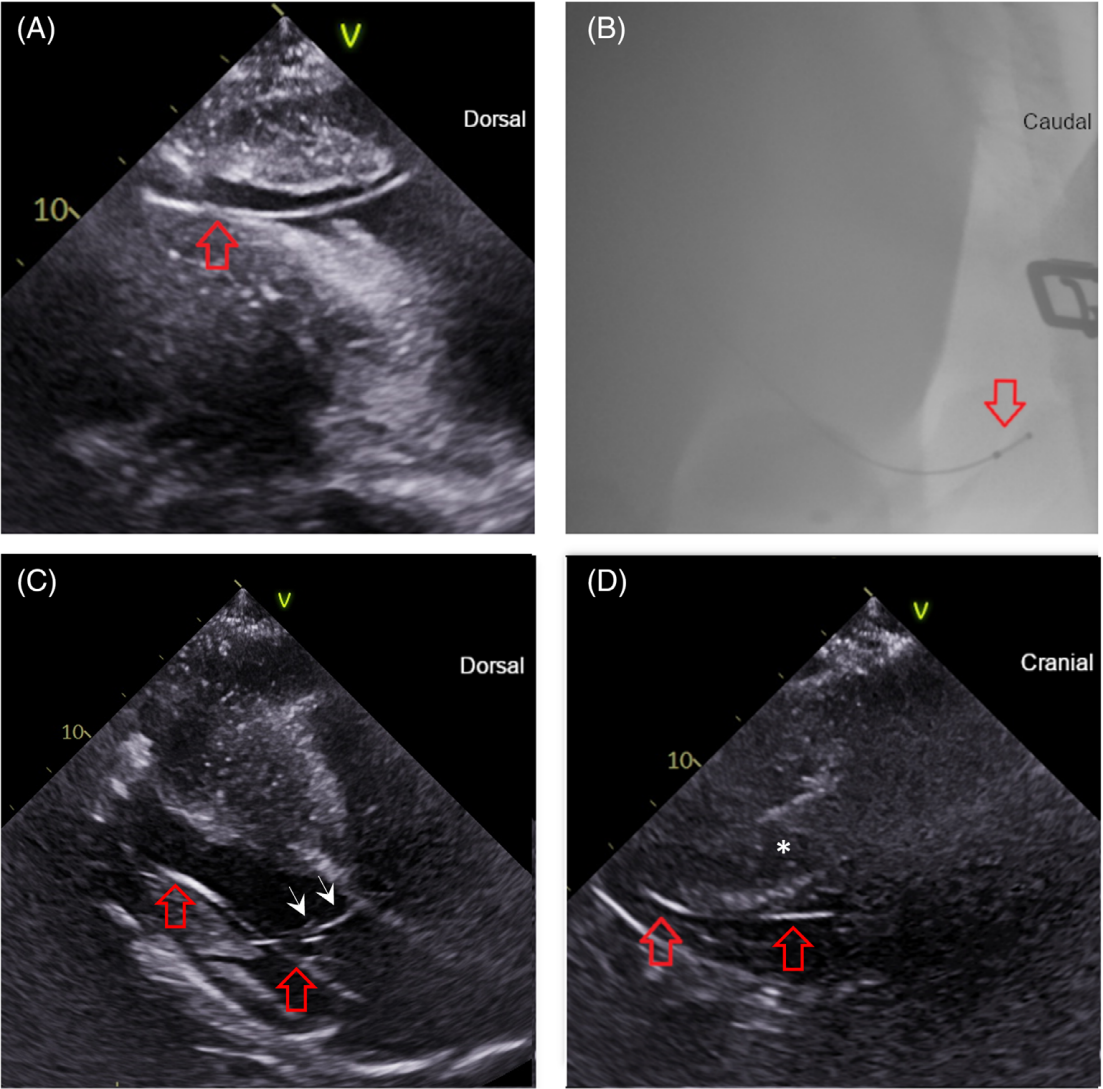
The right ventricle (RV) apex lead position in selected echocardiographic and fluoroscopic images. The lead in each image is denoted by the red arrow(s). (A) Echocardiographic right parasternal long‐axis modified 4‐chamber image acquired from the fifth intercostal space to highlight the lead tip going toward the RV apex. Dorsal is to the right of the image. (B) Fluoroscopic right lateral image showing the lead tip curved in caudal RV apex. Cranial is to the left of the image. (C) Echocardiographic left parasternal long‐axis image showing the lead going through the tricuspid valve (white arrow heads) into the RV. Dorsal is to the right of the image. (D) Left parasternal short axis view after the lead in 3C as it extends caudally into the RV apex. The interventricular septum is denoted by an asterisk. Cranial is to the right of the image.

**FIGURE 4:**
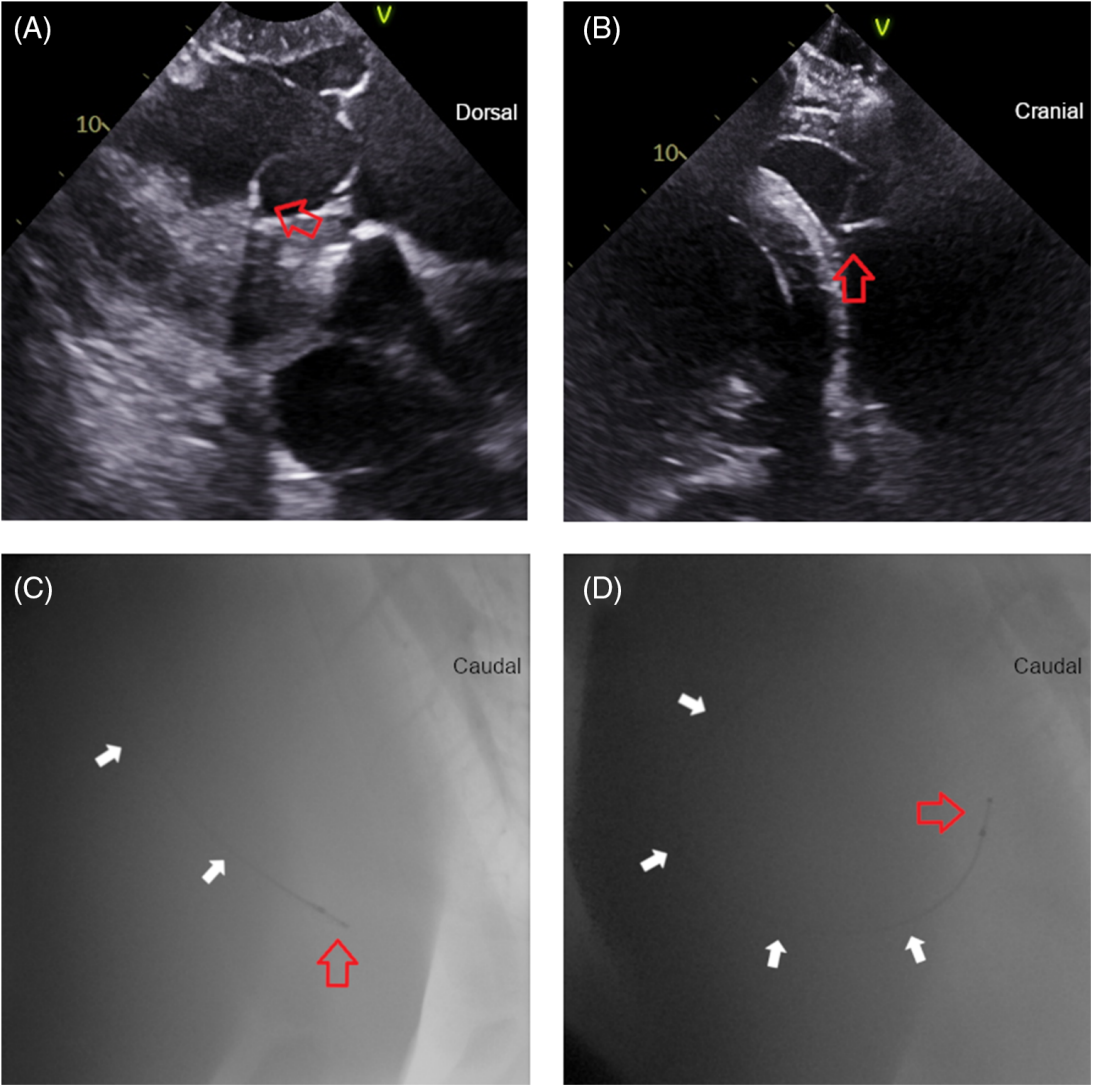
The mid‐right ventricle (RV) lead position in selected fluoroscopic and echocardiographic images. The lead is denoted by the white arrows and lead tip by the red arrow. (A) Echocardiographic right parasternal long‐axis modified left ventricular outflow tract view showing the lead tip angled toward the interventricular septum. Dorsal is to the right of the image. (B) Right parasternal short axis view showing the lead tip extending toward the interventricular septum in the RV mid ventricle position. (C,D) Fluoroscopic right lateral images with the lead straight in the mid RV (C) and curved caudally under the tricuspid valve (D). Cranial is to the left of the images.

**FIGURE 5:**
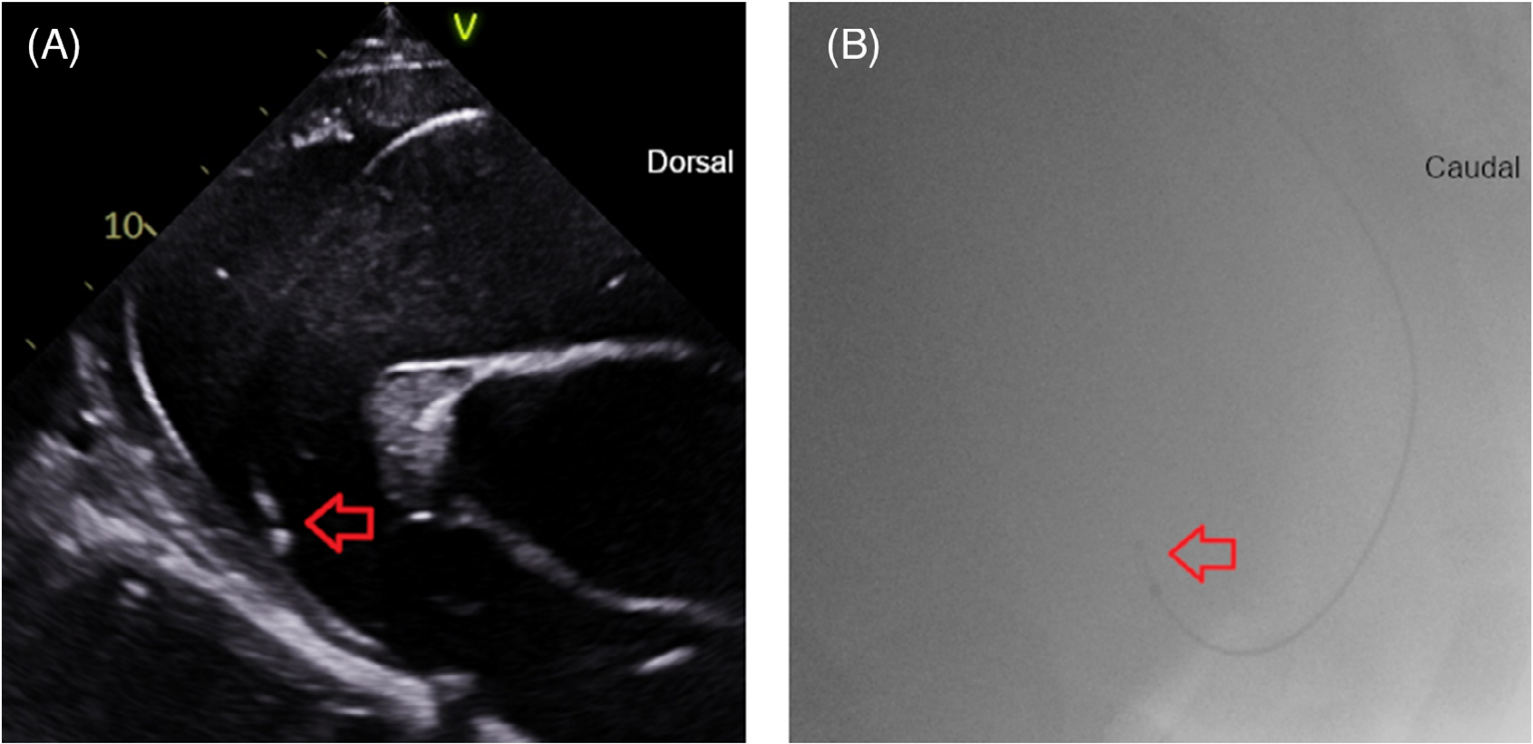
The right ventricular outflow tract lead position in selected echocardiographic and fluoroscopic images. The lead tip is denoted by the red arrow. (A) Echocardiographic right parasternal long‐axis view of the right ventricular outflow tract. Dorsal is to the right of the image. (B) Fluoroscopic right lateral image. Caudal is to the right of the image.

**FIGURE 6:**
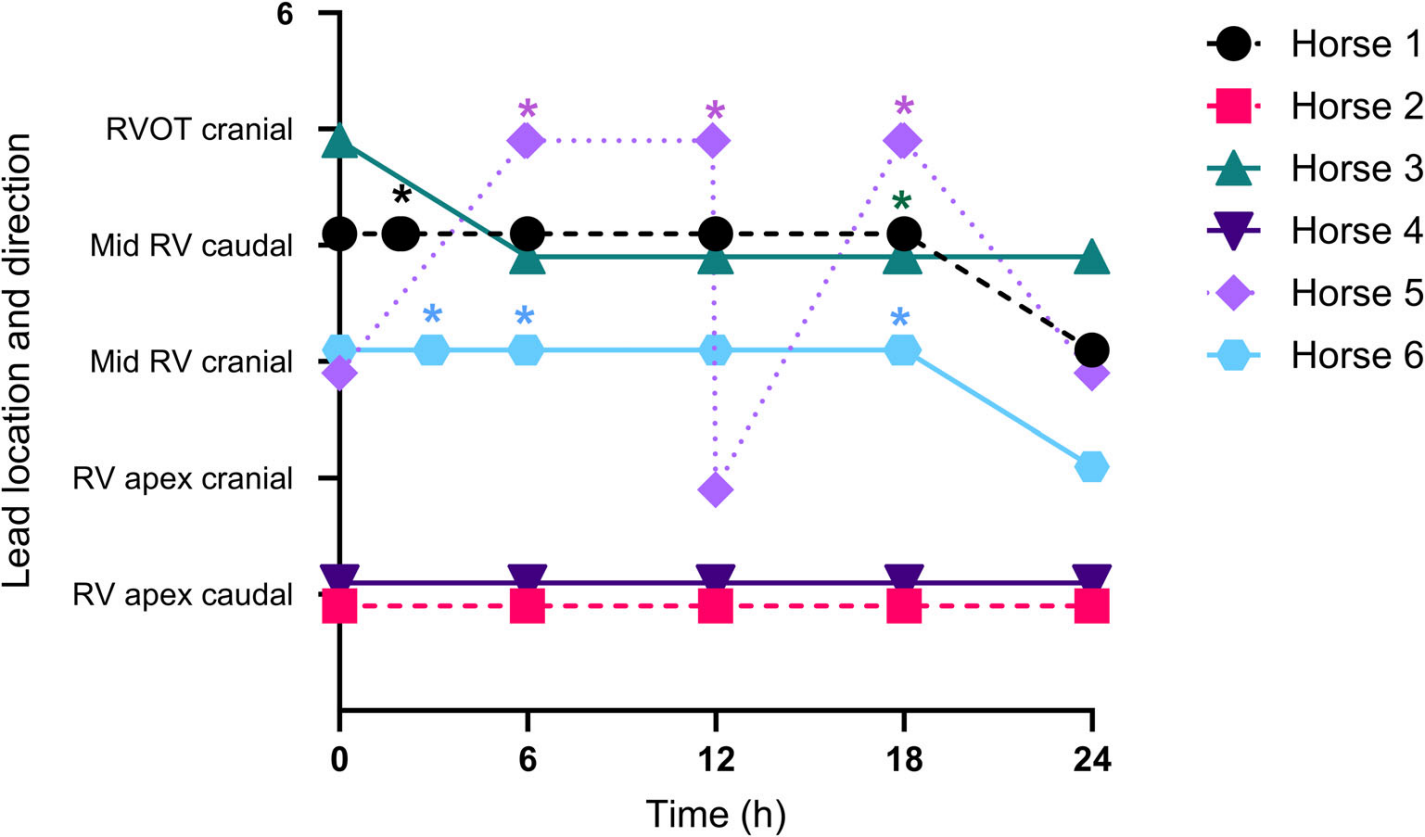
Echocardiographic lead position and configuration in each horse over the 24 h unrestricted period in a stall. Stall‐side repositioning of the pacing lead is marked by an asterisk. RV, right ventricle; RVOT, right ventricular outflow tract.

At the start of the 24 h unrestricted period, lead positions were RV apex (*n* = 2, Figure 3, Videos 1 and 6, 7, 8), mid RV (*n* = 3, Figure 4, Videos 2 and 9), and RVOT (*n* = 1, Figure 5, Videos 3 and 10). The configurations were 3 angled cranially and 3 angled caudally. The 2 horses with leads located in the RV apex angled caudally remained in that position and configuration throughout the study period with no repositioning necessary. Leads in the remaining 4 horses changed position and/or configuration during the unrestricted pacing period and required repositioning in the stall with echocardiographic guidance between 1 and 3 times in each horse (Figure 6).

**VIDEO 1:** Fluoroscopic right lateral video showing the lead tip curved caudally in the RV apex.

**VIDEO 2:** Fluoroscopic right lateral video of the mid right ventricle (RV) position.

**VIDEO 3:** Fluoroscopic right lateral video showing the lead tip in the right ventricular outflow tract (RVOT).

**FIGURE 7:**
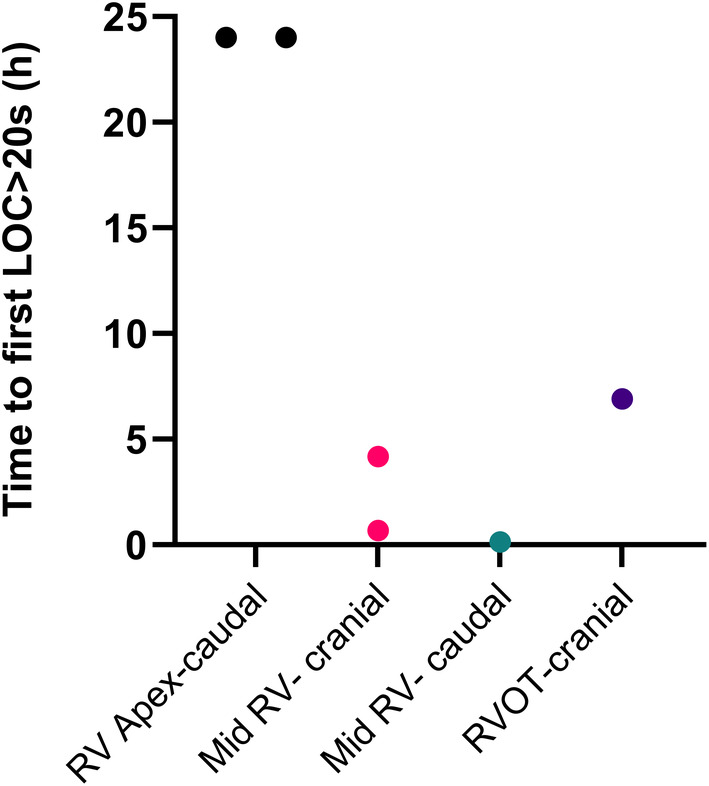
Time to LOC >20 s in hours for the leads of each individual horse during the entire 24 h unrestricted period based on lead position in both fluoroscopic and echocardiographic images at the start of the 24 h unrestricted period. A time to LOC >20 s of 24 h indicates no loss of capture during the study period. RV, right ventricle; RVOT, right ventricular outflow tract.

**FIGURE 8:**
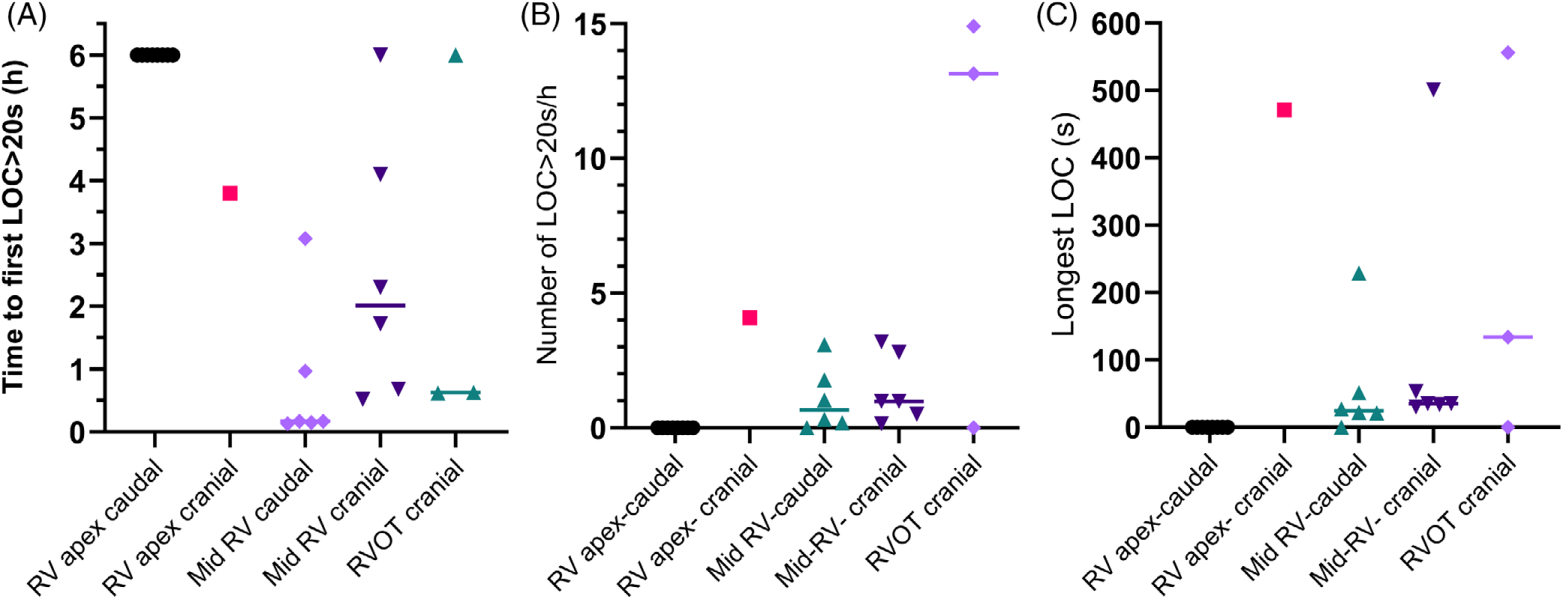
Echocardiographic lead positions and configurations and measures of pacing success for each of the 6 h checkpoints. (A) Time to first loss of capture >20 s (LOC > 20 s); a longer time represents a more successful lead position. Leads in the caudal RV apex had a longer time to LOC > 20 s than all other positions combined *P* < 0.001. (B) Number of LOC > 20 s periods per hour; a lower number represents a more successful lead position. (C) Longest LOC in seconds; a lower number represents a more successful lead position. Leads in the caudal RV apex had a lower longest LOC than all other positions combined *P* = 0.019. RV, right ventricle; RVOT, right ventricular outflow tract.

Time to LOC > 20 s over the entire unrestricted pacing period is shown in Figure 7. The 2 horses with pacing leads angled caudally in the RV apex were successfully paced throughout the 24 h period. The remaining 4 horses had time from placement to first LOC > 20 s ranging from 0.1 to 6.9 h, number of LOC > 20 s/h ranging from 1.0 to 1.8 and longest LOC from 53 to 556 s. When considering measures of pacing success at each 6 h checkpoint (0–6, 6–12, 12–18, and 18–24 h, Figure 8), leads located in the caudal RV apex had longer time to first LOC > 20 s compared with leads in the other locations combined (5.9 (5.1–6.6) h vs. 2.0 (0.6–3.4) h, *P* < 0.001). The length of the longest period of LOC was lower for leads in the caudal RV apex position compared with other locations (0.0 (0–0) vs. 126 (41–210) s, *P* = 0.019). The number of LOC > 20 s/h did not differ significantly between leads located in the caudal RV apex (0 (0–0)) and those in other locations (3.9 (0–8.3)).

Median pacemaker output was 5.0 [5–6.3] mA, with a median capture threshold of 1 [0.6–3.0 mA]. The median sensitivity setting was 2.0 [1.4–2.5] mV, with a median sensitivity threshold of 2.5 [1.5–4.0] mV. When adjusted for placement modality, capture threshold was lower with the pacing lead located in the RV apex compared with all other locations (1.4 (0.7–2.2) mA vs. 2.3 (2.1–2.5) mA, *P* = 0.04).

QRS widths and peak amplitude in channels 1, 2, and 3 in the different lead locations are shown in Table 1. The QRS morphologies in channels 1, 2, and 3 for each of the different lead positions are shown in Figure S3. “Q” morphology was most common in channel 1 for leads in all positions. “rS” and “Q” morphologies were most common in channel 2 for leads in all positions. “S” morphology was present in channel 2 for a small number of RVOT positions only. In channel 3, “qR” morphology was more frequently found with leads located in the RV apex, whereas “R” morphology was more frequently found with leads located in the RVOT.

**TABLE 1:** Electrocardiographic variables with echocardiographically determined lead positions and configurations.

Echocardiographic lead position	RV apex cranial	RV apex caudal	Mid‐RV cranial	Mid RV caudal	RVOT cranial
Channel 1—peak amplitude (mV)	−0.7 [−1.3 to −0.7]	−1.3 [−1.4 to −1.0]	−0.6 [−0.8 to −0.6]	−1.4 [−1.6 to −0.9]	−0.9 [−1.1 to −0.7]
Channel 1—QRS width (ms)	101 [85 to 121]	107 [99 to 109]	97 [94 to 102]	117 [95 to 125]	101 [85 to 108]
Channel 2—peak amplitude (mV)	−3.5 [−3.7 to −1.7]	−3.7 [−3.2 to −2.9]	−2.6 [−2.0 to −1.9]	−2.3 [−3.6 to −1.5]	−2.5 [−3.3 to −1.9]
Channel 2—QRS width (ms)	141 [111 to 156]	143 [127 to 156]	119 [112 to 126]	143 [120 to 147]	131 [123 to 156]
Channel 3—peak amplitude (mV)	2.7 [1.3 to 2.8]	2.3 [2.1 to 2.4]	1.8 [1.2 to 2.2]	1.5 [0.4 to 2.4]	1.0 [1.3 to 2.5]
Channel 3—QRS width (ms)	125 [118 to 155]	145 [131 to 157]	122 [115 to 129]	132 [127 to 138]	141 [133 to 155]
	*n* = 3	*n* = 12	*n* = 9	*n* = 8	*n* = 4

*Note*: Data are reported as median [IQR] and acquired from both placements and all 6 h checkpoint in each horse (*n* = 6/horse).

Abbreviations: ms, millisecond; mV, millivolt.

No complications requiring intervention occurred in any of the horses during the study period. One horse developed a 0.7 x 0.7 cm fibrin clot on the lead tip that did not cause clinical signs or necessitate treatment. All horses experienced early ventricular beats, none of which required treatment. Plasma cardiac troponin I samples were within normal limits (<0.07 ng/mL) at all timepoints. Echocardiograms and 24 h ECGs performed 1–3 months after pacing lead placement were unremarkable. Fluoroscopic and echocardiographic images from both placements and repositioning were categorized retrospectively in an unblinded manner; however, agreement was not reached on lead position in 13% of timepoints assessed and on configuration in 15% of timepoints assessed. There was agreement on fluoroscopic and echocardiographic position and configuration in all placements before the start of the 24 h pacing period.

## 4 DISCUSSION

This study demonstrates medium term (24 h) TV‐TP is feasible in ambulatory, stall confined, unsedated horses. A technique for lead placement with both primary echocardiograhic and standing fluoroscopic guidance is described. Use of a secondary imaging modality resulted in repositioning in 50% of placements regardless of primary modality, and was perceived to be beneficial in optimizing position in the RV apex. There were no clinically significant complications. Leads were maintained in the RV for the 24 h period with varying degrees of intermittent LOC. The RV apex lead position was associated with better capture, however, achieving this position was challenging with the 6F pacing leads used.

Identification of the RV apex as the optimal lead position is consistent with reports in other species.^1^, ^5^, ^7^, ^9^, ^39^, ^40^ However, despite multiple attempts, the RV apical position was not achieved in some horses because of difficulties manipulating lead direction. The use of more rigid, steerable pacing leads and/or longer, flexible introducer sheaths may improve the ability to achieve a desired position in the caudal RV apex. In horses where the RV apex position was not achieved and there was frequent LOC, stall side lead repositioning was performed with echocardiographic guidance. Even without repositioning, LOC was almost always intermittent with frequent loss and spontaneous resumption of capture associated with movement. Therefore, the primary factor driving the decision to reposition was to reduce the frequency of LOC, but additional considerations included movement of the lead tip on echocardiography, under sensing (resulting in early paced beats), and high capture thresholds. Pacing leads contained within sterile coverings and secured with Tuohy‐Borst adapters facilitated repositioning by enabling aseptic lead movement without the need for re‐suturing.

During the 24 h monitoring period, the 4 horses with leads in the mid‐RV and RVOT experienced LOC > 20 s therefore did not meet the criteria for successful pacing. A LOC > 20 s was considered clinically significant based on previous reports^24^, ^27^ and the authors' clinical experience. However, the length of LOC which results in syncope in individual equids is unknown and likely variable depending on factors including severity of the underlying bradyarrhythmia, rate, and presence of an escape rhythm, cardiac demand, acuteness of onset, and heart rate immediately before the LOC.^14^ Therefore, with close monitoring, all lead positions in the current study may have been sufficient to avoid collapse and/or cardiopulmonary arrest.

Lead dislodgement and loss of capture also presents an issue with both TV‐TP and permanent pacing leads in human and canine patients.^6^, ^10^, ^12^, ^41^ Management includes movement restriction and constant telemetric ECG monitoring.^6^, ^39^, ^40^ Larger cardiac size is hypothesized to be associated with lead dislodgement in large breed dogs and therefore may also be a factor in the horse.^14^ Operator inexperience is also associated with lead dislodgement.^10^, ^12^ All horses in the present study were free to move unrestricted in their stalls and the operators had little experience with pacing lead placement. Therefore, it is conceivable that interventions, which restrict or limit horse movement, such as sedation or cross‐tying, and greater operator experience may improve the success of TV‐TP in horses. ROM studies were performed before the unrestricted pacing period with the aim of identifying and addressing unstable lead placements. Lead movement and LOC during ROM after 3 placements suggested that although ROM can help identify unstable lead placements for repositioning, consistent capture during ROM does not predict unrestricted pacing success. All horses in the present study had consistent capture on ROM before the 24 h unrestricted pacing period.

This is the first study to describe pacing lead placement using standing fluoroscopic guidance in the horse. Overall, lead positions were distributed similarly after primary echocardiographic and fluoroscopic guidance. During fluoroscopy, personal protective equipment was worn and total radiation exposure for the study was below the annual occupational limit. The perceived advantages of fluoroscopy were rapid visualization of pacing lead location in a single image (Videos 4 and 5), the ability to examine extracardiac structures (useful to identify loops in the jugular vein and pacing lead locations in the azygous vein), and shorter placement times. Conversely, echocardiography enabled visualization of the position of the pacing lead relative to intracardiac landmarks and avoids radiation exposure. Standing robotic fluoroscopy is not widely available and poses challenges with equipment safety in horses with symptomatic bradyarrhythmias. Therefore, while the authors' perception was that concurrent use of both modalities optimized lead placement, in clinical cases fluoroscopy may be better used as an adjunct to verify pacing lead position once the horse is stabilized. Traditional radiographs could also serve this purpose, without the ability for real‐time guidance. In horses with symptomatic bradyarrhythmias, rapid onset of pacing and personnel safety are also important considerations. Initiating pacing as the lead passes through the tricuspid valve could facilitate earlier onset of pacing thus reducing the risk of syncope. The use of a guidance sheath or steerable pacing lead may enable the lead to be more readily directed into the RV apex also reducing placement time.

**VIDEO 4:** Fluoroscopic right lateral image following the path of the pacing catheter as it enters the heart and curls to be angled cranially in the right ventricle apex approaching the right ventricular outflow tract.

**VIDEO 5:** Fluoroscopic right lateral image showing the pacing lead being rotated under fluoroscopic guidance to change from angled cranially to angled caudally and advanced into the caudal right ventricle apex.

**VIDEO 6:** Echocardiographic left parasternal long axis view focused on the lead passing through the tricuspid valve and coursing toward the RV apex.

**VIDEO 7:** Echocardiographic left parasternal short axis loop corresponding to the more apical portion of the lead in Video 6 as it courses ventrally and caudally to the RV apex.

**VIDEO 8:** Echocardiographic right parasternal long axis view from the fifth intercostal space demonstrating the lead coursing toward the RV apex.

**VIDEO 9:** Echocardiographic right parasternal long axis view focused on the lead in the mid RV curving toward the interventricular septum.

**VIDEO 10:** Echocardiographic right parasternal right ventricular outflow tract view showing the lead in the RVOT position.

Despite nonblinded assessment of fluoroscopic and echocardiographic images, there remained some locations in which agreement over lead position and configuration was not reached. Therefore, further work is needed to validate intracardiac catheter locations on standing fluoroscopy. Lead configuration (cranial vs. caudal) was more readily determined by fluoroscopic images. The location of the lead tip within the RV was more readily determined by echocardiographic images. In addition to the described echocardiographic views, imaging from a left parasternal long axis view focused on the RV apex was helpful to identify the lead tip within the RV apex (Figure 3C,D).

In addition to imaging guidance, capture threshold and ECG morphology are used to evaluate pacing lead positioning in humans^2^, ^3^, ^6^, ^9^, ^42^ and dogs.^10^ In this study, capture threshold was lowest with the lead located in the RV apex with a median threshold of 0.8 mA and the ECG had a consistent “qR” morphology in channel 3. This is similar to the target threshold of <1 mA in people,^5^, ^43^ and further supports the RV apex as the optimal placement location. Trends or abrupt changes in pacing thresholds may indicate reduced myocardial contact suggestive of increased risk of LOC. Therefore, monitoring pacing thresholds may be useful to guide repositioning decisions in clinical cases.^2^, ^39^ Intracavitary^5^, ^9^, ^15^ and surface electrocardiograms^5^ have been described to aid pacing lead placement in other species. This method often requires concurrent use of another modality and extensive electrophysiologic knowledge.^5^ Determining QRS morphology at different pacing lead locations was not a primary objective of this study, but there were subjective differences in the QRS morphology with different lead positions. Changes in ECG morphology within an individual patient may be useful as an indicator of change in lead position. Further work assessing ECG morphology in different lead locations, utilizing recently developed lead systems for equine electrocardiography^44^ and vectorcardiography^45^, ^46^ may provide additional information.

No clinically significant complications occurred in this study. Complication rates from TV‐TP in humans vary from 6% to 60%.^2^, ^3^, ^42^, ^47^ There is minimal information on the complications of TV‐TP in dogs, however complication rates from permanent pacing are 12%–55%.^10^, ^12^, ^41^ Reported complications include failure of capture, infection, myocardial puncture, cardiac perforation/hemopericardium, life‐threatening arrhythmias, air embolism, arterial puncture, significant hemorrhage, diaphragmatic pacing, and venous thrombosis.^2^, ^3^, ^40^, ^42^, ^47^, ^48^, ^49^

Short duration, self‐limiting ventricular ectopy occurred during lead placement, as in other species where it rarely requires intervention.^3^, ^8^, ^10^, ^42^ However, significant ventricular arrhythmias do rarely occur^41^ so telemetric monitoring and antiarrhythmic preparedness are indicated. Ventricular ectopy fell into 2 categories: (1) paced complexes that were premature relative to a preceding sinus beat. These were hypothesized to be the result of poor myocardial contact/excessive lead movement impairing the sensing of intrinsic sinus beats, or inappropriate (too high) sensitivity settings on the pacemaker, and (2) ventricular ectopy that was not associated with pacemaker discharge, hypothesized to be triggered by physical contact of the pacing lead with the myocardium. In clinical patients, interventions, if needed, include verifying appropriate pacemaker settings (sensitivity, output, and rate) and repositioning to improve lead contact. On many occasions, the pacemaker sensed T waves when at the recommended sensitivity setting of half threshold value, likely because of prominent T waves in equids. Therefore, the sensitivity settings were often set higher than 50% of threshold value. This may have contributed to undersensing and inappropriate pacing. As there is no detriment to sensing the T waves beyond slowing rate, if early paced complexes become a concern, lower sensitivity settings may be preventative even in the presence of T wave sensing.

## 5 LIMITATIONS

This study consisted of a small number of healthy horses, therefore, does not confirm efficacy in horses with clinical bradyarrhythmias. It is assumed that horses with clinically significant bradyarrhythmia will respond to pacing in a similar manner, however the effect of temporary cardiac pacing on any intrinsic or escape rhythms in horses remains to be determined. Additionally, the objective of this study was to assess the feasibility of the technique rather than compare guidance methods; therefore, further investigations using a cross‐over design are necessary to establish the optimal guidance modality.

The difficulty in placing the lead in the target location of the RV apex was unanticipated thus resulting in unanticipated variations in lead location between horses. Measures of pacing success in the different lead positions were assessed retrospectively with the small numbers available. A randomized trial with greater number of horses and confirmation of lead location is necessary to properly evaluate the effect of lead position on pacing success and ECG morphology. Additionally, measures of pacing success were made relative to lead position at the start of each 6 h observation period. However, in 8/24 instances, the leads changed position between the beginning and end of the observational period. Therefore, data on pacing success is only truly indicative of lead position at the start of the 6 h observation period. Each 6 h observation point was treated as an individual data point for statistical analyses. This decision may have introduced a degree of pseudoreplication. Despite these limitations, the authors believe this comparison provides useful preliminary information regarding the relationships between lead position and pacing success.

Antimicrobial prophylaxis was used in 50% of the horses in the study because of initial concerns regarding maintenance of sterility while performing a novel technique. There was no evidence of infection in the 50% of horses that did not receive antimicrobial prophylaxis, which is consistent with recommendations in the human literature for medium term TV‐TP.^5^, ^47^ However, further work with a larger number of horses is needed to confirm this finding and decisions regarding antimicrobial prophylaxis should also take into consideration likelihood of subsequent permanent pacemaker implantation in which scenario they would be recommended.^11^, ^12^

## 6 CONCLUSIONS

In conclusion, 24 h TV‐TP is feasible in horses using both echocardiographic and fluoroscopic guidance for lead placement. It has potential to stabilize horses with symptomatic bradyarrhythmias over the medium term. Leads positioned caudally in the RV apex paced most consistently. However, this position can be challenging to achieve; stiffer steerable leads/guidance sheaths may help improve placement. Continuous telemetric ECG monitoring is also recommended to facilitate rapid repositioning if LOC occurs. TV‐TP leads could also be of benefit in horses considered at increased risk of developing symptomatic bradyarrhythmias with sedation and/or general anesthesia. Further work is necessary to refine the technique particularly readily achieving a catheter position in the RV apex, confirming optimal lead position, and establishing target thresholds values.

## CONFLICT OF INTEREST STATEMENT

Authors declare no conflict of interest.

## OFF‐LABEL ANTIMICROBIAL DECLARATION

Procaine penicillin (22 000 IU/kg IM q12h) and gentamicin (8 mg/kg IV q24h) were used off label as prophylaxis.

## INSTITUTIONAL ANIMAL CARE AND USE COMMITTEE (IACUC) OR OTHER APPROVAL DECLARATION

Approved by the Ethical Committee of the Veterinary School of University of Pennsylvania (IACUC #806884).

## HUMAN ETHICS APPROVAL DECLARATION

Authors declare human ethics approval was not needed for this study.

## Supporting information


**Supplementary Figure S1.** (A) The robotic fluoroscopy platform where pacing leads were placed. (B) The pacing lead secured with a Tuohy‐Borst Adapter. (C) The non‐sterile neck wrap made from stockinette and secured to the leather surcingle with brown gauze. The telemetric ECG and pacemaker are shown attached to the leather surcingle.

**Supplementary Figure S2.** Percentage of each QRS morphology in each of the modified telemetric ECG leads 1, 2, and 3 for each echocardiographic location using data collected from each of the 6 h checkpoints: right ventricular apex (RV Apex), mid right ventricle (Mid RV) and right ventricular outflow tract (RVOT).

**Supplementary Table S1.** Individual horse variation in technique.
