# Supplementary figures and images for: Twenty‐four hour continuous transvenous temporary right ventricular pacing in healthy horses
Source: J Vet Intern Med. 2024 Mar 21;38(3):1751–64. doi: 10.1111/jvim.17027 (PMC11099695; doi:10.1111/jvim.17027)

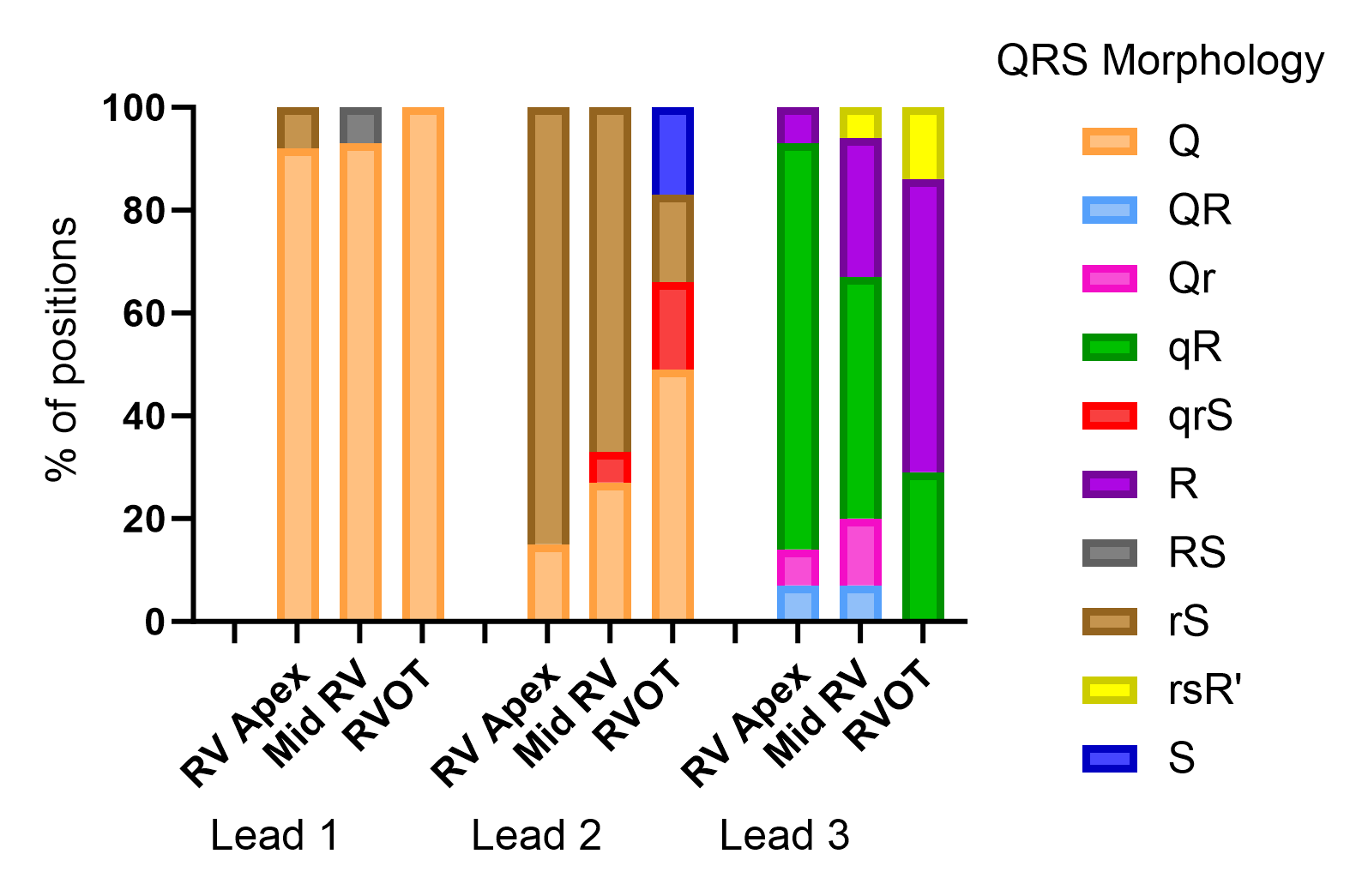

Supplement: Supplementary file 2 — Supplementary Figure S2. Percentage of each QRS morphology in each of the modified telemetric ECG leads 1, 2, and 3 for each echocardiographic location using data collected from each of the 6 h checkpoints: right ventricular apex (RV Apex), mid right ventricle (Mid RV) and right ventricular outflow tract (RVOT). [file JVIM-38-1751-s002.tif]
