# Supplementary material for: Twenty‐four hour continuous transvenous temporary right ventricular pacing in healthy horses
Source: J Vet Intern Med. 2024 Mar 21;38(3):1751–64. doi: 10.1111/jvim.17027 (PMC11099695; doi:10.1111/jvim.17027)
Supplement: Supplementary file 3 — Supplementary Table S1. Individual horse variation in technique. [file JVIM-38-1751-s003.pdf]

Supplementary Table 1: Individual horse variation in technique

Horse	Introducer Size	First Pacing Catheter	Second Pacing Catheter
1	7 F	6 F torque-directed	6 F torque-directed*
2	10 F	6 F torque-directed*	6 F torque-directed
3	7 F	5 F balloon floatation	6 F torque-directed*
4	7 F	6 F torque-directed*	6 F torque-directed
5	7 F	6 F torque-directed*	6 F torque-directed
6	10 F	6 F torque-directed*	6 F torque-directed

* indicates which catheter was in place for the 24-hour unrestricted pacing period
